# Bacteriological Spectrum and Antibiotic Susceptibility on Blood Culture in Newly Diagnosed Pediatric Patients With Acute Lymphoblastic Leukemia During the Induction Phase

**DOI:** 10.7759/cureus.25470

**Published:** 2022-05-30

**Authors:** Usman Fawad

**Affiliations:** 1 Department of Pediatric Hematology/Oncology, The Children’s Hospital and Institute of Child Health, Multan, PAK

**Keywords:** staphylococcus aureus, e. coli, bacterial isolates, blood culture, acute lymphoblastic leukemia

## Abstract

Background

Acute lymphoblastic leukemia (ALL) is the most common cancer diagnosed in children worldwide. This study was conducted to find out the trends in the bacteriological spectrum and antibiotic susceptibility on blood culture in newly diagnosed children with acute lymphoblastic leukemia during the induction phase at a pediatric oncology unit in South Punjab, Pakistan.

Methodology

This cross-sectional study was conducted from January 1, 2020, to June 30, 2021. A total of 263 newly diagnosed ALL cases of both genders aged up to 16 years were included. Adopting full aseptic measures, the blood samples of all children were sent for culture and sensitivity testing to the institutional laboratory immediately after collection on the eighth day of the induction phase in all children. Bacterial isolates and their sensitivity/resistance patterns were noted.

Results

Out of 263 children with ALL, 172 (65.4%) were males. Overall, the mean age was 7.4±3.4 years (ranging between 1 and 16 years). B-cell type was the commonest type noted in 204 (77.6%) children. Out of a total of 52 cases with positive blood culture findings for bacterial isolates, there were 28 (53.8%) cases with gram-negative bacterial isolates, while 24 (46.2%) were gram-positive bacterial isolates. *Escherichia coli* *(E. coli)* was the commonest type of gram-negative bacteria noted among 18/28 (64.3%) cases, while *Staphylococcus aureus (S. aureus) *was the most frequent gram-positive bacterial isolates in 13/24 (54.2%). We found meropenem, linezolid, clindamycin, piperacillin, tazobactam, and amikacin to have the highest antimicrobial sensitivities, while commonly adopted antibiotics such as ciprofloxacin, cefotaxime, cefoperazone, amoxiclav, and ampicillin were found to have high resistance rates.

Conclusion

Gram-negative bacterial isolates formed the majority of the positive blood culture cases. *Escherichia coli*, *Staphylococcus aureus*, and *Klebsiella pneumonia* *(K. pneumonia) *were the most common types of bacterial isolates. Routinely used antibiotics such as ciprofloxacin, cefotaxime, cefoperazone, and ampicillin were found to have high rates of resistance.

## Introduction

Acute lymphoblastic leukemia (ALL) is the most common cancer diagnosed in children worldwide. Although no population-level statistics are available from Pakistan, it is estimated that the annual incidence of ALL is 3.7-4.9 per 100,000 children in the United States [1]. Infection is considered to be the commonest cause of treatment-related morbidity and mortality in children with the diagnosis of cancer globally [2,3]. Among children with ALL, infections are noted to be one of the major reasons behind increased rates of mortality in developing countries. Compared to developed countries, infection-related mortality soars up to 10-fold among children with ALL in developing countries [4]. Timely identification and treatment of causative agents can significantly improve the survival rates among pediatric cancer patients [2]. Ward et al. who estimated global childhood cancer survival rates and priority settings revealed that supporting services and care including infection control have the highest survival gains [5].

Geographical variations exist regarding infection-related mortality and outcomes even among different settings of the same geographical locations. These differences could be attributed to differences in the epidemiology of causative agents and variations in inaccessibility to resources thought to prevent, identify, and treat these infections [6]. Due to these differences, it is very difficult to implement a single approach to reduce morbidity and mortality among children with cancer. A study from India analyzing bacterial spectrum and sensitivity patterns of pathogens among patients of febrile neutropenic patients with hematological malignancies found *Escherichia coli (E. coli)* to be the commonest pathogen involved in 43% of culture-positive cases [7]. Another study from the United States evaluating children with newly diagnosed acute leukemia notedcoagulase-negative* *staphylococci (CoNS) as the most commonly involved microorganism (12/29, 41.4% cases) [6].

In Pakistan, not much work is seen regarding the pattern of bacterial isolates and their antibiotic susceptibility among children with ALL. It was imperative to design a study on the subject to fill up the much-needed gap and provide useful information about the most common causative agents and their sensitivity/resistance patterns to most commonly used antibiotics. The present study was conducted to find out trends in the bacteriological spectrum and antibiotic susceptibility on blood culture in newly diagnosed pediatric patients with ALL during the induction phase at a pediatric oncology unit in Southern Punjab, Pakistan.

## Materials and methods

This cross-sectional study was conducted at the Department of Pediatric Oncology, The Children’s Hospital and Institute of Child Health, Multan Pakistan, from January 1, 2020, to June 30, 2021. Approval from the Institutional Ethical Committee was acquired (approval number: CHC/IEC/20-148). Informed and written consent was sought from parents or guardians or children.

A total of 263 newly diagnosed ALL children of both genders aged up to 16 years were included, which gives a reasonable power to the study considering the rare nature of this disease. The induction phase of chemotherapy lasts 28 days (day 8 to day 35), starting from day 8 of therapy following the prophase. Adopting full aseptic measures, the blood samples of all children were sent for culture and sensitivity testing to the institutional laboratory immediately after collection on the eighth day of the induction phase in all children. Laboratory tests were done at the institutional laboratory. The blood sample was taken by a nurse or a medical technician and immediately sent to the laboratory following the standard protocols of using the blood collection tube. Blood culture was performed using the traditional method for bacterial identification. The blood sample (5 mL) was mixed with *Trypanosoma* broth with a 1:9 ratio and incubated at 37°C. Signs of bacterial growth were checked routinely. Blood agar, chocolate agar, and MacConkey agar were used for subculture. In the case of positive blood culture, bacterial identification was made using colony characteristics, the gram reaction of the organism, and biochemical tests as per standard guidelines. The isolation of one or more known bacteria was observed from blood culture. The isolated bacteria were *Escherichia coli (E. coli)*,* Staphylococcus aureus (S. aureus)*,* Klebsiella pneumoniae (K. pneumoniae)*,* Pseudomonas aeruginosa (P. aeruginosa)*,* Salmonella typhi (S. Typhi)*,coagulase-negative* *staphylococci(CoNS),* *and* Enterococcus faecalis (E. faecalis)*. An antimicrobial susceptibility test was performed on all blood cultures isolated by disk diffusion. About 3-5 colonies of bacteria were transformed into a tube containing 5 mL sterile normal saline and gently mixed with a homogeneous suspension. The incubated tubes were left to dry, and a set of the antibiotic disc was then placed. The commonly used disc was meropenem, amikacin, piperacillin-tazobactam, gentamycin, clindamycin, vancomycin, ceftriaxone, ciprofloxacin, imipenem, linezolid, cefoperazone, amoxiclav, amoxicillin, clarithromycin, cefotaxime, and levofloxacin. A standard treatment schedule was adopted and adhered to during the induction phase. A special proforma was formed to record all study data.

For data analysis, the Statistical Package for Social Sciences (SPSS) version 26.0 (IBM Corp., Armonk, NY, USA) was utilized. Quantitative data such as age were represented as means and standard deviations (SD). Qualitative data such as gender, area of residence, types of ALL, bacterial isolates, and their sensitivity/resistance patterns were shown as frequencies and percentages.

## Results

In a total of 263 newly diagnosed ALL cases, there were 172 (65.4%) males. Overall, the mean age was 7.4±3.4 years (ranging between 1 and 16 years), while 116 (44.1%) children were aged above 5-10 years. The majority of the children (147 (55.9%)) belonged to urban areas. B-cell type was the commonest type of ALL noted among 204 (77.6%) children. Blood culture studies reported positive findings for bacterial isolates in 52 (19.8%) children, while seven (2.7%) had an existence of fungus. Table 1 shows the characteristics of all children.

**Table 1:** Characteristics of Children With Acute Lymphoblastic Leukemia

Characteristics		Number (%)
Gender	Male	172 (65.4%)
	Female	91 (34.6%)
Age (Years)	<5	93 (35.4%)
	>5 to 10	116 (44.1%)
	>10	54 (20.5%)
Area of Residence	Urban	147 (55.9%)
	Rural	116 (44.1%)
ALL Types	B-Cell	204 (77.6%)
	T-Cell	52 (19.8%)
	Mixed Type	7 (2.7%)
Major Symptoms	Fever	72 (27.4%)
	Cough	51 (19.4%)
	Dyspnea	38 (14.4%)
	Diarrhea	26 (9.9%)
Blood Culture for Isolates	Bacterial Isolates	52 (19.8%)
	Fungus	7 (2.7%)
	Negative	204 (77.6%)

Out of a total of 52 cases with positive blood culture findings for bacterial isolates, there were 28 (53.8%) gram-negative bacterial isolates, while 24 (46.2%) were gram-positive bacterial isolates. *Escherichia coli* was the commonest type of gram-negative bacteria noted among 18/28 (64.3%) cases, while *Staphylococcus aureus* was the most frequent gram-positive bacterial isolates found in 13/24 (54.2%). Table 2 shows the antibiotic sensitivity and resistance patterns concerning bacterial isolates among children with ALL.

**Table 2:** Antibiotic Sensitivity and Resistance Patterns With Respect to Bacterial Isolates Among Children With Acute Lymphoblastic Leukemia

Bacterial Isolate	Most Sensitive Antibiotics	Sensitivity (%)	Most Resistant Antibiotics	Resistance (%)
*Escherichia coli (E. coli)* (n=18)	Meropenem	100	Ampicillin	89
	Piperacillin Tazobactam	94	Cefoperazone	83
	Amikacin	89	Amoxiclav	83
*Staphylococcus aureus (S. aureus) *(n=13)	Clindamycin	100	Amoxicillin	69
	Gentamycin	92	Ciprofloxacin	69
	Vancomycin	85	Ampicillin	62
*Klebsiella pneumoniae (K. pneumoniae)* (n=9)	Meropenem	89	Clarithromycin	67
	Ceftriaxone	89	Amoxiclav	67
	Gentamycin	78	Cefotaxime	67
*Pseudomonas aeruginosa (P. aeruginosa)* (n=5)	Ceftriaxone	80	Gentamycin	60
	Ciprofloxacin	80	Ampicillin	60
	Amikacin	60	Piperacillin Tazobactam	40
*Salmonella typhi (S. typhi)* (n=3)	Meropenem	100	Ampicillin	67
	Imipenem	100	Levofloxacin	67
	Ceftriaxone	67	Ciprofloxacin	67
Coagulase-negative staphylococci (CoNS) (n=2)	Amikacin	100	Ceftriaxone	50
	Meropenem	100	Gentamycin	50
	Piperacillin Tazobactam	50	Ciprofloxacin	50
*Enterococcus faecalis (E. faecalis)* (n=2)	Vancomycin	100	Ciprofloxacin	100
	Linezolid	100	Gentamycin	100
	Clindamycin	50	Ampicillin	50

## Discussion

It is vital to periodically determine the most commonly involved organisms and their antimicrobial sensitivities in a particular population to enhance the clinical approach and treatment among children with ALL [8-10]. The present study is the first of its kind to analyze trends in the bacteriological spectrum and antibiotic susceptibility on blood culture in newly diagnosed pediatric patients with ALL during the induction phase at a pediatric oncology unit in South Punjab, Pakistan. We noted that the majority of the children with newly diagnosed ALL were male. A study by Burns et al. from the United States noted that 51.5% of children with newly diagnosed acute leukemia are male [6]. Local data from Lahore analyzing children with ALL observed that 79.2% of cases were male [11]. Another study from Karachi found that 66% of ALL cases were male [8]. In this study, we noted that B-cell was the most common type of ALL as noted among 77.6% of ALL cases. B-cell ALL is the most common type of ALL, accounting for more than 70% of ALL cases; therefore, our findings were consistent with what has been reported in the literature [11].

In the present study, blood culture studies reported positive findings for bacterial isolates in 19.8% of children. The proportion of children with positive blood culture findings in the present study was less than what was reported in a recent study conducted by Rajeswari et al. who analyzed infections during the induction phase of children with ALL, where they found that 30.6% of the children have microbiologically confirmed infection [12]. Gram-negative bacterial isolates were noted in 53.8% of positive cases. Data from India in a recent study revealed that 80% of ALL cases with culture-positive findings for bacterial isolates have gram-negative organisms [12]. A study from China also revealed that 59.9% of cases with positive blood culture findings have a gram-negative bacterial presence [13].

We also noted that *E. coli* (gram-negative) was the most common bacterial isolate present in 34.6% of newly diagnosed ALL cases, followed by *Staphylococcus aureus* (gram-positive), which was found in 25% of cases. A study from Qatar by El-Mahallawy et al. observed that among 268 children with microbiologically confirmed bloodstream bacterial infections, coagulase-negative* *staphylococci were the commonest bacterial isolates noted in 19.8% of cases, followed by *Staphylococcus aureus* (16.4%) and *Acinetobacter *spp. (8.2%) [14]. Data from China found *Pseudomonas aeruginosa* to be the most common bacterial isolates noted in 11.6% of acute leukemia cases [13]. All these data further emphasize that significant differences exist in the distribution of the most commonly involved etiological agents among children with ALL, so frequent analysis of the most commonly involved pathogens must be conducted to obtain first-hand knowledge about the trends of the most prevalent microorganisms involved [15-18].

We found meropenem, linezolid, clindamycin, piperacillin, tazobactam, and amikacin to have the highest antimicrobial sensitivities, while commonly adopted antibiotics such as ciprofloxacin, cefotaxime, cefoperazone, amoxiclav, and ampicillin were found to have high resistance rates. Ampicillin, cefotaxime, and amoxiclav are commonly used antibiotics, but we noted high rates of resistance, which demands a reevaluation of these treatment options. A study conducted in Bangladesh observed that ciprofloxacin was effective against all bacteria, and it helped in reducing infections [17]. However, in the present study, we found that ciprofloxacin had the highest resistance to *S. aureus*, coagulase-negative staphylococci, and *E. faecalis* and maximum sensitivity to *P. aeruginosa*. In our study, ampicillin had resistance to nearly all bacteria excluding *K. pneumoniae*. In addition to ampicillin and ciprofloxacin, amoxicillin also had resistance to *S. aureus*. A study conducted in Pakistan by Irfan et al. observed that no resistance was found for imipenem and meropenem [19]. In our current study, we observed that vancomycin had the highest antimicrobial sensitivity against *E. faecalis*. Effective control of infections is directly linked to the survival of children with acute leukemia, so our findings reinforce that antibiotics should be used judicially adopting the right dosage and duration while there is a need to revise local protocols for the treatment considering a change in the pattern of commonly present causative agents [20-23]. Some researchers have reported other factors such as prolonged antibiotic exposure, previous hospitalization, and prophylactic antibiotic usage for the development of antibiotic resistance [24-27].

Our study had some limitations as well. As this was a single-center study, our findings cannot be generalized. We were unable to identify risk factors associated with bacterial infections. Being a cross-sectional study, we could not document the outcome and follow-up data in the current set of patients, which would have given us valuable insight.

## Conclusions

Gram-negative bacterial isolates formed the majority of the positive blood culture cases.* Escherichia​​​​​​ coli*, *Staphylococcus aureus*, and *Klebsiella pneumonia* were the most common types of bacterial isolates. Routinely used antibiotics such as ciprofloxacin, cefotaxime, cefoperazone, and ampicillin were found to have high rates of resistance.

There is a need for continuous surveillance of the spectrum of locally prevalent pathogens and their susceptibility patterns that can surely help in formulating therapeutic options among newly diagnosed ALL patients.
